# A Single-Step Purification and Molecular Characterization of Functional Shiga Toxin 2 Variants from Pathogenic *Escherichia coli*

**DOI:** 10.3390/toxins4070487

**Published:** 2012-06-25

**Authors:** Xiaohua He, Beatriz Quiñones, Stephanie McMahon, Robert E. Mandrell

**Affiliations:** Western Regional Research Center, U.S. Department of Agriculture, Agricultural Research Service, 800 Buchanan Street, Albany, CA 94710, USA; Email: beatriz.quinones@ars.usda.gov (B.Q.); stephanie.mcmahon@ars.usda.gov (S.M.); robert.mandrell@ars.usda.gov (R.E.M.)

**Keywords:** cell-free translation assay, cytotoxicity, purification of Shiga toxins, Shiga toxin 2 variants, thermal stability of Shiga toxins

## Abstract

A one-step affinity chromatography method was developed to purify Shiga toxin 2 variants (Stx2) Stx2a, Stx2c, Stx2d and Stx2g from bacterial culture supernatants. Analysis of the purified Stx2 variants by denaturing gel electrophoresis revealed 32 kDa and 7 kDa protein bands, corresponding to the Stx2A- and B-subunits, respectively. However, native gel electrophoresis indicated that purified Stx2c and Stx2d were significantly higher in molecular weight than Stx2a and Stx2g. In a cytotoxicity assay with Hela cells, the 50% cytotoxic dose of Stx2a and Stx2g were 100 pg and 10 pg, respectively, but 1 ng each for Stx2c and Stx2d. Interestingly, analysis of the 50% inhibitory dose in a cell-free translational system from rabbit reticulocyte lysates indicated that Stx2g had a lower capacity to inhibit protein synthesis than the other Stx2 variants. The cytotoxicities in Hela cells were neutralized with an anti-Stx2B antibody and were denatured at 80 °C for 1 h. These findings demonstrated that Stx2 variants exhibited different toxicities, holotoxin structure, and stabilities using distinct systems for assessing toxin activities. The development of a simple method for purification of Stx2 variants will enable further studies of Stx2-mediated toxicity in various model systems.

## Abbreviations

(CD50): 50% cytotoxic dose
(HUS): Hemolytic uremic syndrome
(ID50): 50% inhibitory dose
(LB): Luria-Bertani
(mAb): Monoclonal antibody
(PBS): Phosphate-buffered saline
(PAGE): Polyacrylamide gel electrophoresis
(Stx): Shiga toxin
(STEC): Shiga toxin-producing *Escherichia coli*
(SD): Standard deviation

## 1. Introduction

Shiga toxin (Stx)-producing *Escherichia coli* (STEC) is a frequent cause of severe human diseases including bloody diarrhea and hemolytic uremic syndrome (HUS) [1,2]. Stxs are thought to play a prominent role in the pathogenesis of STEC infections. There are two types of Stxs produced by STEC strains, Stx1 and Stx2 [3]. Both types of Stxs are encoded by *stx* genes on temperate bacteriophages [4] and have an AB_5 _structure, in which a single A-subunit is associated with five identical B-subunits. The A-subunit has a molecular weight of 32 kDa and is an active component of the Stx and functions as an *N*-glycosidase. It inhibits protein synthesis by cleavage of an adenine base from the 28S rRNA component of the eukaryotic ribosomal 60S subunit, resulting in cell death [5]. Each B-subunit has a molecular weight of 7.7 kDa and contains multiple binding sites for the natural Stx receptors globotriaosyl ceramide (Gb_3_) [6] or globotetraosyl ceramide (Gb_4_) [7]. These receptors located on the surface of mammalian cells are responsible for binding the Stx and its internalization by endocytosis. Despite their structure similarities of Stx1 and Stx2, they exhibit significant differences in biological activity. Recent epidemiological and molecular typing studies indicate that STEC strains producing Stx2 have been associated more closely with the severe human disease conditions HUS and hemorrhagic colitis than STEC strains producing Stx1 [8,9]. Nucleotide and amino acid sequence analyses have revealed three distinct Stx1 variants, Stx1a, Stx1c, and Stx1d [10,11,12,13]. In contrast to the Stx1 variants, a diverse and heterogeneous group of seven distinct variants of the Stx2 have been identified [14,15,16,17,18,19,20]. These variants differ from each other in terms of their affinity for host receptors, cytotoxicity, animal pathogenicity, and response to activation by intestinal mucus [21,22]. There has been considerable confusion in the nomenclature of Stx2 typing. In this study, we use the nomenclature proposed in 2009 at the 7^th^ International Symposium on Shiga Toxin-Producing *E. coli* Infections in Buenos Aires, designating seven Stx2 subtypes as Stx2a, Stx2b, Stx2c, Stx2d, Stx2e, Stx2f, and Stx2g [23,24]. The Stx2a, Stx2b, Stx2c and Stx2d variants are reported most frequently as causing human illness [25,26]. Stx2e is associated primarily with the edema disease of swine [17] and is rarely isolated from humans [27,28]. Stx2f has been isolated from feral pigeons [19], but STEC strains harboring Stx2f were recently reported to cause human illness [29]. Sequence analysis revealed that Stx2e and Stx2f display the most divergence from Stx2a at the amino acid level. Stx2g was identified from a bovine strain of *E. coli* O2:H25 and exhibited the highest DNA sequence homology with Stx2a and Stx2c [20]. It has been reported that routine PCR and serological assays were not able to detect all Stx2 subtypes because of the differences in the specificities of stx PCR primers or anti-Stx antibodies for the various Stx subtypes [30]. The expanding number of Stx2 variants discovered and their subtle differences in DNA and encoded amino acid structures emphasize the need to have pure, or at least partially pure, Stxs and specific anti-Stx antibodies for immunodiagnostic assays and to investigate the role of each Stx2 variant in the pathogenesis of human diseases. However, there are limited amounts of purified Stx2 available commercially (limited to the Stx2a type only) because of select agent regulations of the US Centers for Disease Control and Prevention and no Stx2 variants toxin stocks are available commercially to date. This led us to evaluate methods for purification of Stx2 variants. We describe in this study a simple, rapid method for purification of four Stx2 variants and characterize their purity, quantity and maintenance of biological activity of these Stx2 variants purified using this method. Differences were revealed in holotoxin structure, stability, cytotoxicity, and enzymatic activity among these toxin preparations.

## 2. Materials and Methods

### 2.1. Sample Preparation

Pure bacterial culture supernatants were prepared from the strains listed in Table 1 as described previously [31]. The *stx2* variant genes expressed by STEC strains were subtyped by PCR using sequence-specific primers as described in Table 2. All strains were negative for *stx1* variants by PCR using sequence-specific primers as described in Table 2. PCR reagents were supplied by Promega Corporation (Madison, WI) and PCR primers were purchased from Eurofins MWG Operon (Huntsville, AL). As template for the PCR reaction, bacterial crude lysates were prepared as described in previous studies [32]. PCR amplifications were performed in a 25 µL reaction mixture, each containing 5 µL of the bacterial crude lysate, 0.5µM of each primer and 12.5 µL of 2× GoTaq^®^ Green Master Mix (Promega Corporation). The reaction mixtures were placed in a Dyad Peltier Thermal Cycler (Bio-Rad Laboratories, Hercules, CA), and amplifications were performed with the conditions described in Table 2. Amplified products were analyzed in 2% agarose gels containing 0.04 µL/mL GelRed Nucleic Acid Stain (Phenix Research, Candler, NC, USA). 

**Table 1:** Characteristics of Shiga toxin (Stx)-producing *Escherichia coli* (STEC) strains used for purification of Stxs.

Strain	Other names	Serotype	*stx* genotype^b^	Origin^d^	Reference
RM10638		O157:H7	*stx2a*	Cow (2009)	This study
RM7005	EH250	O118:H12	*stx2b.*	Clinical	[16]
RM10058		O157:H7	*stx2c.*	Bird (2009)	This study
RM8013		ND^a^	*stx2d*	Cow (2008)	This study
RM7110	S1191	O139:NM	*stx2e*	Pig	[17]
RM7007	T4/97	O128:H2	*stx2f*	Feral pigeon	[19]
RM10468		ND^a^	*stx2g*	Cow (2009)	This study
RM4876		O157:H7	*stx*-negative^c^	Watershed (2005)	[33]

^a^ Not determined. ^b ^New *stx* variant subtyping nomenclature as published recently [24,30]. ^c^ The strain is *rfbE*^+^, *eae*^+^, *hlyA*^+^ as determined by methods described previously [34]. ^d^ Year of sample collection is shown in parentheses.

To induce the expression of *stx2*, bacterial cells from single colonies were grown overnight in Luria-Bertani (LB) liquid medium at 37 °C with shaking (250 rpm), and overnight cultures were then diluted in 1:50 LB medium containing mitomycin C (final concentration: 50 ng/mL) and continued to grow for 16 hours at 37 °C. Cells were pelleted by centrifugation at 13,000 × *g* for 10 min at 4 °C, the supernatants were filtered through a 0.45 µm PVDF filter and used for protein purification.

### 2.2. Purification of Stx2

To create a permanent column for affinity purification of Stxs, 3 mg of mouse-monoclonal antibody (mAb) against Stx2 A-subunit (VT135/6-B9 from Sifin Institute, Berlin, Germany) was immobilized through covalent attachment of its primary amines (-NH2) to a 2-mL AminoLink Column using AminoLink Plus Immobilization Kit (Pierce Biotechnology, Rockford, IL) following manufacturer’s instructions in “Procedure for Coupling Protein using the pH 7.2 Coupling Buffer”. 

For affinity purification, filtered bacterial supernatants (200 mL) were concentrated by precipitation at room temperature with saturated ammonium sulfate added to a final concentration of 60%. The precipitate was pelleted by centrifugation at 10,000 × g for 10 min, and then resuspended in 1 mL phosphate-buffered saline (PBS, pH 7.2) at 1:100 dilutions. After desalting using a Zeba Spin Desalting Column (7K MWCO, Pierce Biotechnology), samples containing Stxs were purified by affinity chromatography using the affinity column conjugated with anti-Stx mAb prepared as described above. Briefly, 1 mL of a sample recovered from the desalting column was mixed with an equal volume of PBS buffer and applied to the column. After incubation for 1 h at room temperature, the column was washed with 8–10 mL PBS and the proteins bound were eluted with 8 mL 0.1 M glycine-HCl, pH 2.8 and collected in 2 mL fractions, which were neutralized immediately with 100 µL 1 M Tris-HCl, pH 9.0. The yield of total protein was determined using Pierce 660 nm Protein Assay kit (Pierce Biotechnology). The concentration of Stx in different preparations was determined by ELISA using verotoxin 2 (List Biological Laboratories, Inc., Campbell, CA) as a standard and mAb against Stx2 A-subunit, VT135/6-B9 (Sifin Institute), as a capture antibody and mAb against Stx2 B-subunit, VT136/8-H4 (Sifin Institute), as a detection antibody. These mAbs were specific for Stx2, and no immunoreactivity was observed when tested with purified Stx1. 

### 2.3. Polyacrylamide Gel Electrophoresis (PAGE) and Western Blot

The purity and subunit structure of the Stx was determined by analyzing protein samples in their native and denatured conditions using 4%–12% Native and NuPAGE (denatured) Novex Bis-Tris mini gels (Invitrogen, Carlsbad, CA) following manufacturer’s specifications. All gels were stained with Coomassie Blue G-250 to visualize the proteins. After PAGE, the proteins were electroblotted from a duplicate gel to a PVDF membrane (pore size, 0.45 um; Amersham Hybond-P; GE Healthcare, Buckinghamshire, UK) and Stx on the membrane were detected with anti-Stx mAbs and the Amersham ECL-Plus Western Blotting Detection System (GE Healthcare) following manufacturer’s specifications. The primary mAbs, VT135/6-B9 and VT136/8-H4 (anti-Stx2A and -2B subunits, Sifin Institute), were diluted in PBS, pH 7.3 at 250 ng/mL, and the peroxidase conjugated goat anti-mouse IgG antiserum (Promega Corporation) was diluted to 2 ng/mL for the detection of Stx2 and its variants. 

### 2.4. Cell-Free Translation Assay

The activity of Stx samples was assessed in a cell-free translation assay as described previously, but with small modifications [35]. Serial dilutions of Stxs were added to a translation lysate mixture consisting of nuclease-treated rabbit reticulocyte lysate (Cat. L4960), complete amino acid mixture (1 mM, Cat. L4461), RNasin ribonuclease inhibitor (40 u/µL, Cat. N2111), nuclease-free water (Cat. P1193), and luciferase mRNA (1 mg/mL, L4561) in a ratio (vol/vol) of 35:1:1:36:2, respectively. All reagents were purchased from Promega Corporation. The final ratio of Stx2 and translation lysate mixture was 1:5 (vol/vol). The Bright-Glo Luciferase Assay System was purchased from Promega (Cat. E2620). Black microtiter plates (NUNC 96-well Maxisorp) were purchased from Fisher Scientific Inc. (Pittsburgh, PA). Luminescence was measured as counts per second (cps) in a Victor II plate reader (Perkin Elmer, Shelton, CT). The translation lysate mixture with PBS buffer in lieu of Stx2 was used as a negative control. The enzymatic activity of Stxs was calculated as the percentage of inhibition of protein translation [(cps in negative control − cps in Stx2-treated sample)/cps in negative control] × 100. A 50% inhibitory dose (ID_50_) represents the concentration of Stx necessary to inhibit 50% of protein translation. 

### 2.5. Cytotoxicity of Stx2 and Its Variants

Cytotoxicity assays were modified from a method reported previously by Neal *et al.* [36]. Hela cells (CCL-2) were purchased from American Type Culture Collection (ATCC, Manassas, VA) and cultured in Dulbecco’s Modified Eagle Medium (DMEM, Invitrogen) supplemented with 10% fetal calf serum (Invitrogen) and maintained in a humidified incubator (37 °C, 5% CO_2_). Cells were trypsinized, adjusted to approximately 0.5 × 10^5^ to 1.0 × 10^5^ cells per ml, seeded (100 uL/well) onto 96-well plates (Corning), and allowed to adhere overnight. Hela cells were then treated with Stxs in DMEM at the concentration indicated for 1 h at 4 °C. Cells were washed with DMEM to remove non-internalized Stxs and then incubated for another 24 h at 37 °C and viability was assessed using CellTiter-Glo reagent (Promega) according to the manufacturer’s instructions, except that the reagent was diluted 1:5 in PBS prior to use. Luminescence was measured with a Victor II plate reader (Perkin Elmer). All treatments were performed in triplicate, and the viability of cells grown in medium with PBS (~1 µL) was considered as 100% and used as a negative control (no inhibition on cell growth). The cytotoxicity of Stxs was calculated as the percentage of inhibition of cell growth [(cps in negative control − cps in Stx2-treated sample)/cps in negative control] × 100. A 50% cytotoxic dose (CD_50_) represents the amount of toxin necessary to kill 50% of the attached monolayer of cells in the wells.

### 2.6. Neutralization of Stx Activity by Antibody

Stx was prepared at 5 times the pre-determined CD_50_ concentration and pre-incubated with 50 μg/mL of the mouse mAb, VT136/8-H4 (anti-Stx2 B subunit), in DMEM complete medium at 37 °C for one hour. A 100 μL sample was then added to a 96-well plate containing 0.5 × 10^5^ to 1.0 × 10^5^ actively growing Hela cells/well and incubated at 4 °C for one hour. After washing with fresh DMEM complete medium to remove unbound toxin and antibody the cells were further incubated at 37 °C for another 24 hours before CellTiter-Glo viability analysis as described above. The relative cytotoxic activities of Stx after neutralization were calculated by normalizing the values to the activities of Stxs without neutralization by mAb as 100%. 

### 2.7. Data and Statistical Analyses

All data on enzymatic activity and cytotoxicity of Stxs represent the mean ± standard deviation (SD) of triplicate samples measured in a representative experiment. Three individual experiments were performed. Small variations in results between experiments were observed due to slight changes of Stx activity and other conditions. Statistical differences in toxicity between Stx2 variants were analyzed by ordinary one-way ANOVA followed with Tukey’s Multiple Comparison Test using GraphPad Prism 5 (GraphPad Software Inc., San Diego, CA). The differences were considered significant at *P* < 0.05. 

## 3. Results

### 3.1. Genetic Typing and Sequence Analysis of Stx2 Genes of STEC Strains

Bacterial strains used for the production of Stxs are listed in Table 1. The *stx2* genes present in the STEC strains that were recovered from environmental samples were subtyped by PCR using sequence-specific primers targeting each *stx2* variant (Table 2). Results from the PCR analyses confirmed that only a single *stx2* variant was detected in each STEC strain that was selected for production of Stx2 variants (Table 1). Sequence analysis of *stx2* genes of STEC strains used in this study indicates that the deduced amino acid sequence of Stx2a was identical to its reference strain [16,17,19,37] and the sequences of Stx2c, Stx2d, and Stx2g expressed by environmental STEC strains were highly similar to the published sequences in other strains expressing these Stx2 variants [18,20,38]. A single sequence variation for Stx2c was one amino acid change (from G to S) at amino acid position 124 and for Stx2d was one amino acid change (from R to G) at amino acid position 235. For Stx2g there were two amino acid changes at positions 81 (from V to I) and position 83 (from Q to E). Reference strains were used for the production of Stx2b, Stx2e, and Stx2f [16,17,19]. Table 3 lists the alignment score between Stx2a and other variants based on Clustal 2.0.12 multiple sequence alignments. The deduced amino acid sequences of Stx2c, Stx2d, and Stx2g were highly similar to the sequence of Stx2a (similarity above 94%). However, the sequence of Stx2f is much less similar to the sequence of Stx2a, with homologies of 69 and 82% for the A and B subunits, respectively. 

**Table 2:** PCR primers and conditions for *stx2* variant subtyping.

Target	Primers	Nucleotide sequences (5'→3')	PCR cycle conditions	Ampicon size (bp)	Reference
*stx_1a_*	STX1A F2	CACGTTACAGCGTGTTGCA	94 °C, 2 min (1×); 94 °C 30 s, 52 °C, 1 min,		
	STX1A R2	CGCCCACTGAGATCATCC	72 °C, 40 s (25×); 72 °C, 5 min (1×)	219	[39]
*stx_1c_*	Lin-up	GAACGAAATAATTTATATGT	94 °C, 2 min (1×); 94 °C 1 min, 48.1 °C, 90 s,		
	IOX3	CTCATTAGGTACAATTCT	72 °C, 90 s (30 ×); 72 °C, 5 min (1×)	555	[11]
*Stx_1d_*	VTIA varF	CTTTTCAGTTAATGCGATTGCT	94 °C, 2 min (1×); 94 °C 1 min, 62 °C, 1 min,		
	VTIA varR	AACCCCATGATATCGACTGC	72 °C, 1 min (5×); 94°, 1 min, 58 °C, 1min,		
			72 °C, 1 min (5×); 94°, 1 min, 54 °C, 1min,		
			72 °C, 1 min (20×); 72°, 5 min (1×)	192	[10]
*stx_2a_*	Stx2-F	AGATATCGACCCCTCTTGAA	94 °C, 5 min (1×); 94 °C, 45 s, 60 °C, 45s,		
	Stx2-R	GTCAACCTTCACTGTAAATG	72 °C, 90 s (25×); 72 °C, 7 min (1×)	969	[40]
*stx_2b_*	Stx2-G2-F	TATACGATGACACCGGAAGAAG	94 °C, 5 min (1×); 94 °C, 30 s, 65 °C, 30s,		
	Stx2-G2-R	CCTGCGATTCAGAAAAGCAGC	72 °C, 60 s (25×); 72 °C, 5 min (1×)	300	[41]
*stx_2c_*	Stx2/2c	TTTTATATACAACGGGTA	94 °C, 5 min (1×); 94 °C, 30 s, 51 °C, 30 s,		
	Stx2-G1-R	GGCCACTTTTACTGTGAATGTA	72 °C, 60 s (30×); 72 °C, 5 min (1×)	163	[41,42]
*stx_2d_*	Stx2d-act	CTTTATATACAACGGGTG	94 °C, 5 min (1×); 94 °C, 60 s, 54 °C, 60 s,		
	CKS2	CTGAATTGTGACACAGATTAC	72 °C, 60 s (25×); 72 °C, 5 min (1×)	359	[42]
*stx_2e_*	Stx2-G4-F	CAGGAAGTTATATTTCCGTAGG	94 °C, 5 min (1×); 94 °C, 30 s, 55 °C, 30 s,		
	Stx2-G4-R	GTATTCTCTTCCTGACACCTTC	72 °C, 60 s (25×); 72 °C, 5 min (1×)	911	[41]
*stx_2f_*	Stx2-G3-F	TTTACTGTGGATTTCTCTTCGC	94 °C, 5 min (1×); 94 °C, 30s, 61 °C, 30 s,		
	Stx2-G3-R	TCAGTAAGATCCTGAGGCTTG	72 °C, 60 s (25×); 72 °C, 5 min (1×)	875	[41]
*stx_2g_*	209F	GTTATATTTCTGTGGATATC	94 °C, 5 min (1×), 94 °C, 45 s, 55 °C, 60 s,		
	781R	GAATAACCGCTACAGTA		72 °C, 60 s (25×); 72 °C, 7 min (1×)	573	[20]

**Table 3:** Sequence similarity between Stx2 variants.

Stx2 type	A subunit	Aligned score	B subunit	Aligned score
Stx2a	319 aa	100	89 aa	100
Stx2b	319 aa	93	87 aa	88
Stx2c	319 aa	99	89 aa	96
Stx2d	319 aa	99	89 aa	95
Stx2e	319 aa	94	87 aa	87
Stx2f	319 aa	69	87 aa	82
Stx2g	319 aa	95	89 aa	94

### 3.2. Purification of Stx2 and Stx2 Variants

**Figure 1:**
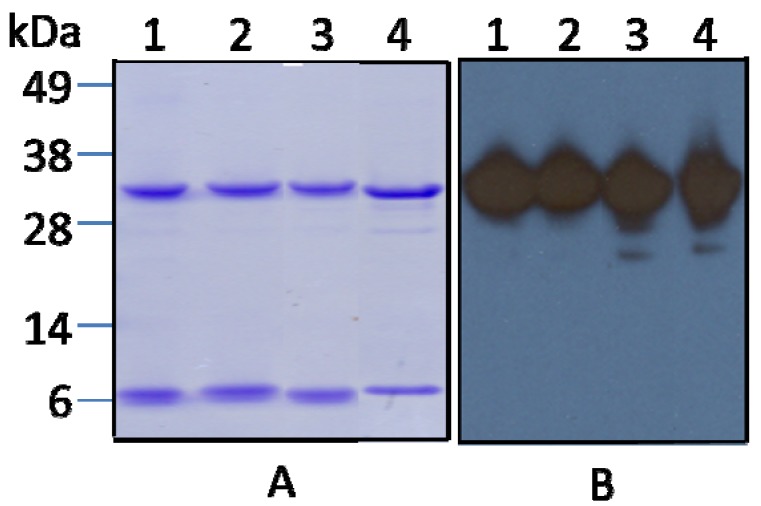
Analysis by SDS-PAGE and Western blot of variant Stx2 proteins in partially purified protein preparations. **(A)** Coomassie stained SDS-PAGE. Lanes 1–4 are 2 μg of protein samples from partially purified Stx2a, Stx2c, Stx2d, and Stx2g preparations, respectively. Molecular markers are indicated as kilodaltons (kDa) at the left side of the panel. **(B)** Western blot of 0.5 μg of Stx as in panel A (lanes 1–4) analyzed with mAb against the Stx2 A-subunit.

**Figure 2:**
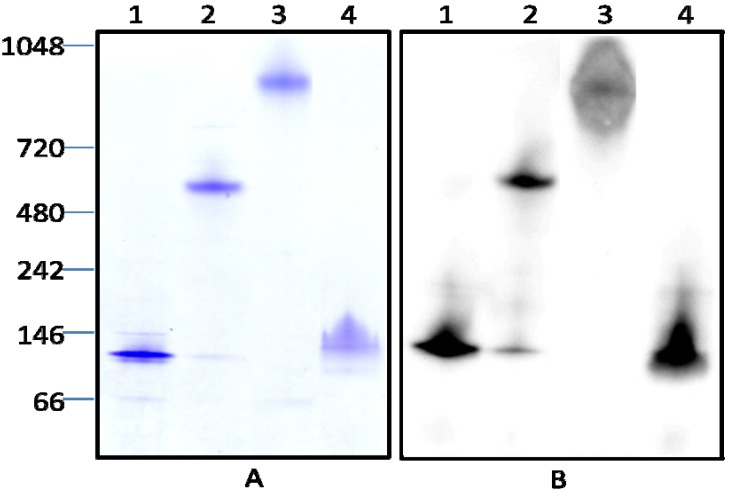
Analysis by Native PAGE and Western blot of Stx2 variants in partially purified protein preparations. **(A)** Coomassie stained SDS-PAGE. Lanes 1–4 are 2 μg of protein samples from partially purified Stx2a, Stx2c, Stx2d, and Stx2g preparations, respectively. Molecular markers are indicated as kilodaltons (kDa) at the left side of the panel. **(B)** Western blot of 0.5 μg of Stx as in panel A (lanes 1–4) analyzed with mAb against Stx2 A-subunit.

To maximize yield and minimize the number of steps required for purification of Stxs, we applied a single one-step purification strategy. Before purification bacterial culture supernatants were first clarified by ammonium sulfate fractionation. The majority of Stx2 protein precipitated at 50%–60% saturated ammonium sulfate (data not shown). After desalting, the proteins from the 50%–60% ammonium sulfate fraction were directly applied to an affinity column conjugated with mAb against Stx2 A-subunit and purified following steps described (see Materials and Methods). The final yields of Stx2a, 2c, 2d, and 2g after affinity purification ranged from 220 to 470 ng/mL of the culture supernatant (Table 4). The amounts of protein recovered from the Stx2b and 2e culture supernatants were low, yielding only 40 and 60 ng/mL, respectively. No proteins were recovered from the Stx2f culture supernatant. Figure 1 shows the analysis of affinity purified Stx2 variants by SDS-PAGE and Coomassie staining using Invitrogen’s NuPAGE Novex Bis-Tris Gel system, following the separation and staining protocols supplied. Two clearly stained protein bands were observed with molecular weights of approximately 32 kDa and 7 kDa, corresponding to the sizes of Stx2 A- and B- subunits in samples from Stx2a, Stx2c, Stx2d, and Stx2g preparations. A lightly stained band with a molecular weight of approximately 27 kDa was found in some of samples, which we speculate is the A_1_ subunit of the toxin (Figure 1A). The identity of the toxin proteins (A and B subunits of the Stx2) was confirmed by Western blot. Figure 1B shows the blot stained with mAb against Stx2A; the blot stained with mAb against Stx2B is not shown. No proteins were detected in the Stx2b, Stx2e, and Stx2f preparations by SDS-PAGE and Western blot (data not shown). This could be caused by a low antibody affinity to these variants or low expression of the strains. Our previous results [31] demonstrated that the toxin activity in supernatant of bacterial strain RM7007 (*stx2f*) was as high as that of *stx2a* strain when tested by a cell-free translation assay, suggesting the expression of *stx2f* by this strain; no protein recovered after affinity purification is most likely due to the weak reactivity of the antibody with the variant Stx2f. While the toxin activities in supernatants of bacterial strains *stx2b* and *stx2e* were only about 60% and 40% of the *stx2a* strain [31], suggesting that the low yields of Stx2b and Stx2e may be due to the combination of low antibody affinity and low expression of these strains. Further characterization was performed for the Stx2a, Stx2c, Stx2d, and Stx2g only. Native PAGE of the toxin preparations revealed that only one major protein band was stained by Coomassie in the samples from Stx2a, 2c, 2d, and 2g preparations and these bands coincided with the bands stained by the mAb against Stx2A in the Western blot analysis (Figure 2). The protein size for the Stx2a and Stx2g was ~72 kDa, as expected for the size of a holotoxin. However, the protein size for the Stx2c was approximately 600 kDa and for the Stx2d was approximately 900 kDa, relative molecular weights much larger than that of their holotoxins. 

**Table 4:** Purification yields of Stx2 variants from bacterial supernatants.

Stx type	Before purification	After purification	Recovery	Cytotoxicity	Enzymatic activity
	(ng/mL of supernatant)	(ng/mL of supernatant)	(%)	(CD_50_)^a^	(ID_50_)^a^
Stx2c	1320	306	23.18	1.00 ng (a)	30 pg/uL (a)
Stx2d	3300	220	6.67	1.00 ng (a)	40 pg/uL (b)
Stx2a	1250	470	37.6	0.10 ng (b)	40 pg/uL (b)
Stx2g	2960	281	9.5	0.01 ng (c)	160 pg/uL(c)
Stx2b	nd^b^	40	nd^b^	nd^b^	nd^b^
Stx2e	nd^b^	60	nd^b^	nd^b^	nd^b^
Stx2f	nd^b^	0	nd^b^	nd^b^	nd^b^

^a^ Differences between numbers with different letters were statistically significant (*P* < 0.05) and between numbers with the same letter were not statistically significant. ^b^ nd, not determined.

### 3.3. Cytotoxicity and Enzymatic Activity of Stx2 Variants

The biological activities of the Stx2 variants purified from this study were assessed by a microtiter cytotoxicity assay using Hela cells. Growing cultures of Hela cells were intoxicated for one hour at 4 °C and continued to be incubated for another 24 h at 37 °C after removing the unbound Stxs. A dose-dependent increase of cytotoxicity was observed for all Stxs at the ranges of concentrations 0.01 to 100 ng/mL (data not shown). The cytotoxic activity of Stx2g purified from strain RM10468 was significantly higher than that of all other Stx2 variants with a CD_50_ value ~10 pg/well. A CD_50 _value of 100 pg was measured for Stx2a from strain RM10638, and a CD_50 _value 1 ng was measured for both Stx2c and Stx2d from strains RM10058 and RM7005, respectively. These findings indicated that Stx2a and Stx2g purified from these strains were more toxic to the Hela cell line used in this study, when compared to Stx2c and Stx2d. There was no significant difference in cytotoxicity between Stx2c and Stx2d using this Hela cell line.

To determine if the appreciable differences in cytotoxicity to Hela cells were due to differences in the catalytic activity of these Stxs, we used a cell-free rabbit reticulocyte lysate assay to monitor the relative inhibition of protein translation by various Stx2 variants. Table 4 indicates that the ID_50_ value of Stx2g was 160 ng/mL, a value significantly higher than the ID_50 _values of Stx2a and other Stx2 variants. These findings suggest that the catalytic activity of Stx2g was lower compared to the other Stx2 variants. These data suggest that differences in cytotoxicities of Stxs to Hela cells are affected by factors other than just the catalytic activity of the Stxs. 

### 3.4. Neutralization of Toxin Activity

**Figure 3:**
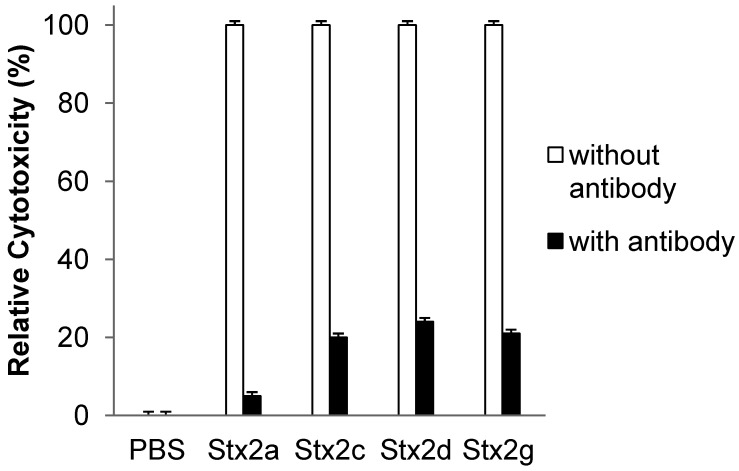
Neutralization of Stx2 variants by mouse mAb (IgG) against Stx2 B-subunit (VT136/8-H4). Stxs at concentrations of 5CD_50_ were pre-incubated with 50 μg/mL of mouse mAb against Stx2B at 37 °C for one hour. The mixture was then added to actively growing Hela cells and incubated at 4 °C for one hour. After removing unbound Stxs, the cells were incubated in fresh DMEM complete medium at 37 °C for another 24 h. The relative cytotoxic activities of the Stxs after neutralization were calculated by normalizing the values to the activities of Stxs without neutralization by mAb as 100%. Results indicate the mean ± SD of three replicates from one representative experiment. Three individual experiments were performed.

To confirm that the purified Stx2 variants were responsible for the cytotoxicity we measured in Hela cells, neutralization tests were performed by pre-incubating Stxs with Stx2-specific mAb against Stx2 B-subunit prior to incubation in the mammalian cells. Figure 3 shows the effect of the Stx2 mAb (VT136/8-H4) on the cytotoxicity of Stx2a, Stx2c, Stx2d, and Stx2g at their corresponding 5CD_50_ concentrations. The relative cytotoxicity of each Stx after neutralization was calculated by normalizing the values to the activities of Stxs without neutralization by mAb as 100%. The cytotoxic activity was reduced 95% for the Stx2a and at least 75% for the Stx2c, Stx2g, and Stx2d after neutralization. Residual cytotoxicities (5 to 25%) were still present even after neutralization. Pre-incubation of Stx2 variants with a non-specific mAb IgG did not affect the toxicities of these Stxs (data not shown). These data suggest that all Stx preparations were active and resulted in cytotoxic activities in Hela cells. At the concentration tested, the mAb, VT136/8-H4, was able to neutralize the majority of the activities of these Stxs. 

### 3.5. Stability of Stx2 Variants

**Figure 4:**
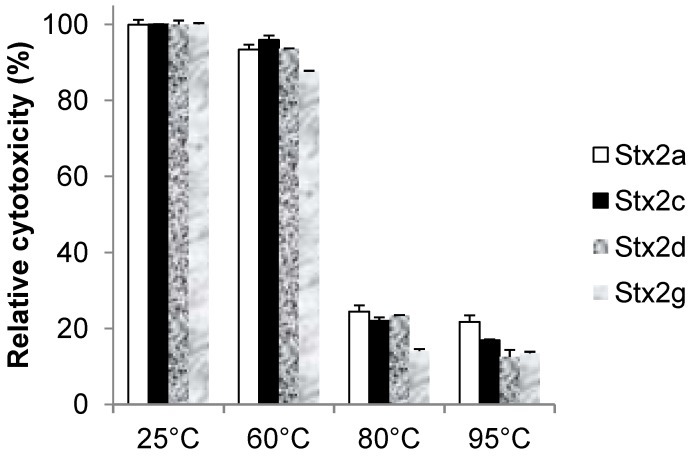
Effect of temperature on Stx2a, Stx2c, Stx2d, and Stx2g cytotoxicity. Partial pure Stxs were heated at the indicated temperature for 1 h. Following heat treatment, residual Hela cell cytotoxicity was determined. The relative cytotoxicity was calculated by normalizing their values to the activity of toxin without heat treatment as 100%. The results represent the mean ± SD of three replicates from one representative experiment. Three individual experiments were performed.

To compare the ability of each Stx to resist conformational changes which may result in loss of cytotoxicity, the thermal stability of biologically active Stx holotoxins were assessed after thermal denaturation. Stxs were incubated for one hour at three different temperature condition and the effects of each temperature on cytotoxicity are shown in Figure 4. A small decrease in cytotoxic activity was observed for all Stxs after heating at 60 °C for one hour and a significant reduction was detected after the Stxs were heated at 80 °C for one hour. The reduction for the Stx2a, Stx2c and Stx2d was between 76% and 78% at 80°, while for the Stx2g was 86%, significant more when analyzed by Tukey’s Multiple Comparison Test (*P* < 0.05). These data suggest that Stx2 variants are relatively stable macromolecules at high temperatures, but as a parameter of holotoxin stability, Stx2g appeared to be relatively less stable than the other three Stx2 variants tested. In order to see if protein degrades after heating, protein samples were analyzed by SDS-PAGE. It was found that protein signals of Stx2 variants on SDS-PAGE decreased with the increase of heating temperature and the signal decrease was proportional to the decrease of their cytotoxicity (data not shown). 

## 4. Discussion

The development of methods for Stx purification will facilitate studies of the mechanisms of the interactions and toxicities of Stxs in humans; it will also help developing antibodies for immunodiagnostic assays. Multiple purification methods for Stxs have been reported previously [23,43,44,45,46], however, these methods require multiple steps, including sample clarification and multiple chromatography steps to separate proteins according to their differences in charge, hydrophobicity and size. We developed a simple method using anti-Stx mAb affinity chromatography to purify Stx2 variants in a single step. Approximately 220–470 ng of Stx2a, 2c, 2d, and 2g were purified from each mL of the culture supernatant tested by this method. In contrast, no, or minimal, Stx could be recovered from bacterial culture supernatants containing Stx2b, Stx2e, and Stx2f. A possible reason for this result is a low affinity of the anti-Stx mAb conjugated to the beads for these three Stx2 variant proteins. This is consistent with the relative lower alignment scores of these variants to the type of Stx2 at the amino acid sequence level and also is in agreement with results reported previously [30]. The Stx preparations obtained by this method appeared to be homogeneous on Coomassie-stained SDS-PAGE (Figure 1). The overall CD_50_ values of Stxs in our experiments were higher than previously reported [43]. The overall yields of Stxs purified by this method were relatively low; ranging from 6.6 to 37.5% of the Stx in the starting material (Table 4). This is due, at least partially, to the low amount of anti-Stx mAb available for preparation of the affinity column due to the high cost of the mAb. However, this approach can be improved with similar and less costly reagents available in the future.

Significant differences in cytotoxic activity to Hela cells were observed between Stx2 variants in the same experimental conditions. The CD_50_ of Stx2g and Stx2a were 10 pg and 100 pg, respectively, whereas, it was 1 ng for the Stx2c and Stx2d. Recent studies have documented a difference in the relative toxicities of Stx2 variants in Vero cells [23,47]. Furthermore, the increased cytotoxicities of Stx2g from STEC strain RM10468 and of Stx2a from STEC strain RM10638 were observed also in an assay with a fluorescent Vero cell line [48]. The factors that contribute to differential cytotoxicities of variant Stx2 proteins warrant further study and purified Stx provide useful reagents for comparing Stx in a variety of assays. These include measuring differences in the receptor binding affinity of the Stx, the specific receptor content of the cell lines used, the amount of inactive Stx and varying degrees of purity in each preparation, as well as structural or functional differences between the Stx molecules. 

Our findings have demonstrated also that differences in holotoxin structure and stability exist between Stx2 variants. Our native PAGE indicates that the sizes of the proteins from the partial pure Stx2c and Stx2d preparations were significantly higher in molecular weight than the proteins from the Stx2a and Stx2g preparations (Figure 2). They were 8 or more times higher in molecular weight than the Stx2 holotoxin and adding a reducing agent to the samples did not change the mobility of these proteins on the native protein gel. We suspect that these slow mobile proteins on the native PAGE were non-covalently bound multimers of the Stx2c and Stx2d holotoxin molecules, because no additional protein bands were observed besides the bands corresponding to the sizes of the Stx2 A-subunit and B subunit on the SDS-PAGE. We also note that the formation of these large Stx complexes in the Stx2c and Stx2d preparations coincided with a lower cytotoxic activity compared with Stx preparations without formation of large Stx complex. It is possible that the formation of Stx multimers may hinder Stx binding to its receptors, resulting in decreased cytotoxicity. This is a result requiring further study. 

The thermal stability of Stx2 reported by several groups has stimulated controversy over interpretation of the results. MacLeod *et al.* reported that cytotoxicity of SLT-IIv (Stx2e) was completely destroyed after heating at 65 °C for 30 min [45]. In contrast, Downes *et al.* [43] and Rasooly *et al*. [49] reported that no major decrease in Stx2 cytotoxicity until the Stx2 was heated at 85 °C or higher. The reason for these dramatically different results is unclear. In our experiments, a significant reduction in cytotoxicity was observed after the Stxs were heated at 80 °C for one hour, moreover, the Stx2g was shown to be more susceptible to high temperature compared to the other Stx2 variants, as indicated by the decrease in cytotoxic activity after heating at 60 °C and 80 °C (Figure 4). Assuming that heat treatment is a suitable test for protein stability, these data suggest that the Stx2g holotoxin is inherently a less stable macromolecule than other variants we tested. The stability differences characterized in the cytotoxicity assay may represent important in vivo differences in intracellular processing or degradation of the Stxs. In our cell-free translation assay, we found that the ID_50_ of Stx2g was much higher than that of other Stx2 variants, indicating that the Stx2g had a lower capacity to inhibit protein synthesis. Given that Stx2g was toxic in Hela cells, but had a low catalytic activity in the cell-free reticulocyte assay, suggests that receptor binding affinities may account for the observed cytotoxicities in Hela cells. Purified Stx2 will facilitate studies of the mechanisms of Stx2 types in different model systems, especially models that can address human disease. 

In summary, we have reported a single-step method for purification of Stx2 produced by STEC strains. Homogeneous Stxs were obtained with high recovery from bacterial culture supernatants of Stx2a, Stx2c, Stx2d, and Stx2g. The Stxs purified using this method exhibited similarities and differences in their holotoxin structure and function. Results obtained from this study will facilitate the development of variant-specific antibodies against Stx2 produced by pathogenic bacteria from human patients and the investigation of the role of Stx2 variants in the pathogenesis of HUS and hemorrhagic colitis.
